# Cnicin as an Anti-SARS-CoV-2: An Integrated In Silico and In Vitro Approach for the Rapid Identification of Potential COVID-19 Therapeutics

**DOI:** 10.3390/antibiotics10050542

**Published:** 2021-05-07

**Authors:** Hani A. Alhadrami, Ahmed M. Sayed, Hossam M. Hassan, Khayrya A. Youssif, Yasser Gaber, Yassmin Moatasim, Omnia Kutkat, Ahmed Mostafa, Mohamed Ahmed Ali, Mostafa E. Rateb, Usama Ramadan Abdelmohsen, Noha M. Gamaleldin

**Affiliations:** 1 Department of Medical Laboratory Technology, Faculty of Applied Medical Sciences, King Abdulaziz University, P.O. BOX 80402, Jeddah 21589, Saudi Arabia; hanialhadrami@kau.edu.sa; 2 Molecular Diagnostic Lab, King Abdulaziz University Hospital, King Abdulaziz University, P.O. BOX 80402, Jeddah 21589, Saudi Arabia; 3 Department of Pharmacognosy, Faculty of Pharmacy, Nahda University, Beni-Suef 62513, Egypt; Ahmed.mohamed.sayed@nub.edu.eg (A.M.S.); hossam.mokhtar@nub.edu.eg (H.M.H.); 4 Department of Pharmacognosy, Faculty of Pharmacy, Beni-Suef University, Beni-Suef 62513, Egypt; 5 Department of Pharmacognosy, Faculty of Pharmacy, Modern University for Technology and Information, Cairo 11865, Egypt; khayrya.youssif@gmail.com; 6 Department of Microbiology and Immunology, Faculty of Pharmacy, Beni-Suef University, Beni-Suef 62511, Egypt; yasser.gaber@pharm.bsu.edu.eg; 7 Department of Pharmaceutics and Pharmaceutical Technology, Faculty of Pharmacy, Mutah University, Karak 61710, Jordan; 8 Center of Scientific Excellence for Influenza Virus, Environmental Research Division, National Research Centre, Giza 12622, Egypt; Yasmin.Moatasim@human-link.org (Y.M.); Omnia.Abdelaziz@human-link.org (O.K.); ahmed_elsayed@daad-alumni.de (A.M.); mohamedahmedali2004@yahoo.com (M.A.A.); 9 School of Computing, Engineering & Physical Sciences, University of the West of Scotland, Paisley PA1 2BE, UK; Mostafa.Rateb@uws.ac.uk; 10 Department of Pharmacognosy, Faculty of Pharmacy, Deraya University, New Minia 61111, Egypt; 11 Department of Pharmacognosy, Faculty of Pharmacy, Minia University, Minia 61519, Egypt; 12 Department of Microbiology, Faculty of Pharmacy, The British University in Egypt (BUE), Cairo 11837, Egypt

**Keywords:** blessed thistle, cnicin, bioinformatics, in silico, SARS CoV-2, MERS CoV, COVID-19

## Abstract

Since the emergence of the SARS-CoV-2 pandemic in 2019, it has remained a significant global threat, especially with the newly evolved variants. Despite the presence of different COVID-19 vaccines, the discovery of proper antiviral therapeutics is an urgent necessity. Nature is considered as a historical trove for drug discovery, especially in global crises. During our efforts to discover potential anti-SARS CoV-2 natural therapeutics, screening our in-house natural products and plant crude extracts library led to the identification of *C. benedictus* extract as a promising candidate. To find out the main chemical constituents responsible for the extract’s antiviral activity, we utilized recently reported SARS CoV-2 structural information in comprehensive in silico investigations (e.g., ensemble docking and physics-based molecular modeling). As a result, we constructed protein–protein and protein–compound interaction networks that suggest cnicin as the most promising anti-SARS CoV-2 hit that might inhibit viral multi-targets. The subsequent in vitro validation confirmed that cnicin could impede the viral replication of SARS CoV-2 in a dose-dependent manner, with an IC_50_ value of 1.18 µg/mL. Furthermore, drug-like property calculations strongly recommended cnicin for further in vivo and clinical experiments. The present investigation highlighted natural products as crucial and readily available sources for developing antiviral therapeutics. Additionally, it revealed the key contributions of bioinformatics and computer-aided modeling tools in accelerating the discovery rate of potential therapeutics, particularly in emergency times like the current COVID-19 pandemic.

## 1. Introduction

**Figure 1:**
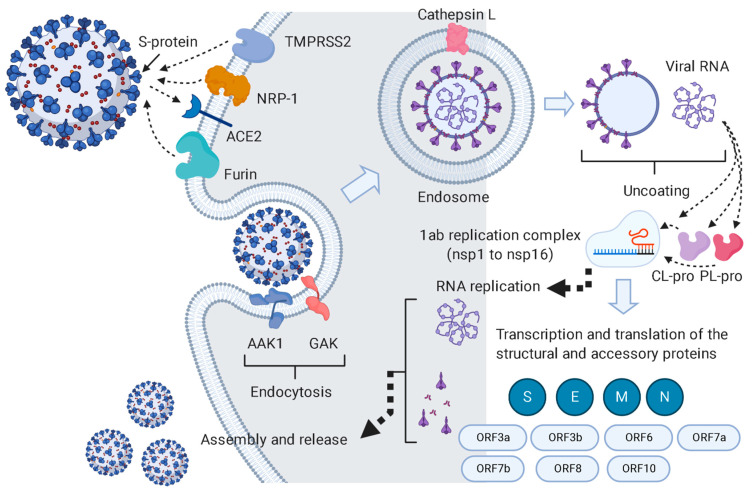
This pathway summarizes the pathogenesis of SARS CoV-2 inside the host cell and indicates the key proteins involved in the viral life cycle. This pathway was constructed with data retrieved from the KEGG website (https://www.genome.jp/kegg-bin/show_pathway?hsa05171+H02398; accessed on 13 December 2020).

SARS CoV-2 (COVID-19) is a newly emerged pandemic triggered by the severe acute respiratory syndrome coronavirus-2 that created an unprecedented global health crisis [1]. It is a zoonotic virus with more highly contagious properties than the Middle East respiratory syndrome virus (MERS CoV) [2]. SARS CoV-2 is of the Coronaviridae family that causes acute respiratory disease, which could be lethal, with an approximate 10.2% mortality rate [1,2]. The disease can cause death due to severe alveolar destruction and hemorrhage, as well as progressive respiratory failure [3]. Coronaviruses are further divided into two subfamilies: Coronavirinae and Torovirinae. The Coronavirinae subfamily is further categorized into four genera—α-, β-, γ-, and δ-coronaviruses—according to the classification of the Worldwide Committee for Logical Classification of Infections [4]. SARS CoV-2 is a positive-sense, single-stranded RNA β-coronavirus that infects mammals and is assumed to have originated from bats [5]. Additionally, it has one of the biggest genomes among other RNA viruses [3,6]. This genome is wrapped with a nucleocapsid protein (N) inside an envelope that consists of three other structural proteins, i.e., the membrane protein (M), the envelope protein (E), and the spike protein (S). The last one (i.e., S protein) gives coronaviruses the outer crown appearance; therefore, the virus is named corona in Latin, meaning the crown [3] (Figure 1). It is also the key protein that mediates the virus entry into the host cell by binding to the host angiotensin-converting enzyme 2 (ACE2) receptors [7,8] (Figure 1). Additionally, other coronaviruses can encrypt an envelope-related hemagglutinin-esterase protein (HE) [3].

The SARS CoV-2 pandemic is a pressing challenge to discover practical approaches and pathways for managing and treating the virus [9]. Moreover, scientists are searching for possible drug targets to develop effective therapeutics to rapidly control this pandemic. The recent findings gave the scientific community a lot of information about SARS CoV-2 structural proteins, as well as those involved in viral nucleic acid replication and other host-specific proteins that have a vital role in the disease pathogenesis [9,10].

Natural products are considered a precious trove of drug leads [11]. Various natural products (e.g., baicalin, ivermectin, and artemisinin) were recently reported as promising SARS CoV-2 inhibitors. They target multiple viral and host-specific proteins involved in viral processing (viral protease), viral entry into host cells, viral replication, and (finally) viral release from infected cells [12]. Moreover, enormous in silico studies were conducted at the beginning of the crisis, and they suggested a wide range of natural products from several chemical classes to be promising anti-SARS CoV-2 agents [12,13].

**Figure 2:**
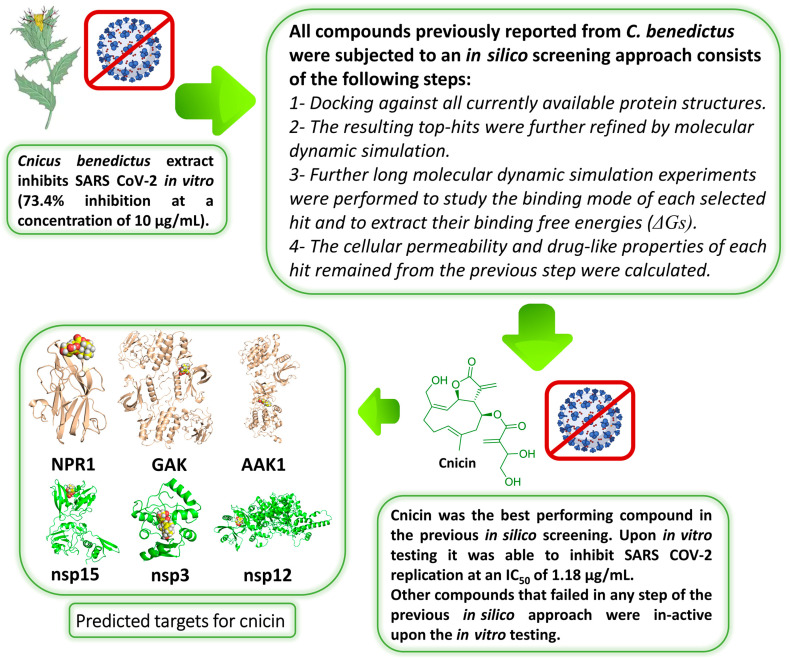
The workflow of the current study.

In this investigation and as a part of our ongoing investigation of natural products for developing new effective anti-COVID-19 therapeutics, we found that the *C. benedictus* extract (CBE) could potentially inhibit viral replication in vitro (produced 73.4% ± 3.2 inhibition at a concentration of 10 µg/mL). This medicinal plant was proven to exhibit a broad-spectrum antimicrobial activity with a good safety profile [14]. Consequently, we conducted an extensive in silico-based investigation by utilizing all of the currently available and well-characterized viral protein targets to determine the main constituents responsible for the CBE’s antiviral activity and, in turn, to explore their in vitro activity. Figure 2 summarizes the strategy applied in the current study.

## 2. Material and Methods

### 2.1. Preparation of the Crude Extract

*C. benedictus* aerial parts were obtained in January 2019 from Faculty of Pharmacy, Minia University, East Desert, Minia, Egypt, and they were authenticated by Prof. AbdelHalim Mohamed, Horticulture Research Institute, Agriculture Research Center, Giza, Egypt. All collected plant materials (0.5 kg) were washed thoroughly, dried, and extracted with 80% ethanol (4 × 500 mL). Subsequently, the resulting liquid extract was dried using a rotary evaporator (IKA™, Hamburg, Germany) to obtain the dried extract, which was kept at 4 °C (CBE). All the other screened plant extracts in our library were produced in the same manner. As a primary and rapid screening, we firstly tested this extract for their cellular cytotoxicity. Non-toxic extracts (with an IC_50_ > 50 µg/mL) were then screened for their %viral inhibitory activity at a fixed concentration (10 µg/mL).

### 2.2. Preparation of C. benedictus’s Pure Compounds

Apigenin 7-*O*-glucoside, astragalin, arctiin, nortracheloside, luteolin, sitogluside, and cnicin were isolated from the CBE according to the previous protocols [14,15,16,17,18]. All of the aforementioned compounds were purchased (Sigma Aldrich, Santacruz Biotech, USA and BioCrick, Sichuan Province, P. R. China) to ensure the antiviral’s maximum possible purity for the in vitro testing.

### 2.3. Data Preparation

#### 2.3.1. Compounds Preparation

Chemical constituents of *C. benedictus* (Figure 2) were identified with an extensive literature search using Google Scholar, PubMed, Research Gate, Web of Knowledge, Reaxys, and Dictionary of Natural Products, as well as the following keywords “*C. benedictus*, isolated compounds, chemical profiling, and bioactive compounds.” The exact isomeric structures of these retrieved molecules were obtained from PubChem [19].

#### 2.3.2. Protein Structures Preparation

Regarding the SARS CoV-2 proteins, all currently available viral and non-viral proteins relevant to COVID-19 were retrieved from the Swiss-Model repository (https://swissmodel.expasy.org/repository/species/2697049, accessed on 2 January 2021) and the String database (https://string-db.org/cgi/covid.pl, accessed on 2 January 2021) [20,21]. All the PDB codes of the proteins used in this study along with their abbreviations and functions are listed in Table S1. The selection criteria were based on the following: (i) the best resolution, (ii) published structure, and (iii) the date of publishing (i.e., the most recent structures were selected). All selected protein structures were prepared according to Charmm force field using the AutoDock Vina software [22].

### 2.4. Ensemble Docking

We used the AutoDock Vina software in all docking experiments [22]. This docking machine uses the Charmm-27 force field for its calculations. All the prepared *C. benedictus* compounds were docked against all of the collected proteins (their PDB codes are listed in Supplementary Materials Table S1). The binding site of each protein was determined according to its co-crystalized ligand. Homology models, along with other proteins without co-crystallized ligands, were subjected to a blind docking protocol, where the software carried out docking on the best druggable sites throughout the protein structure. In this case, we set the search box (i.e., docking box) to enclose the whole protein structure. To account for these proteins’ flexibility, we used their MDS-derived conformers sampled every 10 ns for docking experiments (i.e., ensemble docking) [23]. Subsequently, we ranked the top hits according to their calculated binding energies. We set a docking score of −7 kcal/mol as a cut off to select the best hits. These selected top-hits were subsequently subjected to molecular dynamic simulation experiments to test whether they were able to achieve stable binding over the time of simulation (25 ns). Unstable hits were then excluded. Further long MDS experiments (150 ns) were then performed to study the binding mode of each selected top-scoring compound. Docking poses were analyzed and visualized by the Pymol software [22].

### 2.5. Molecular Dynamic Simulation

MD simulations were performed by Desmond v. 2.2 [24,25], the MDS machine of the Maestro software [26], using the OPLS-AA force field. Proteins systems were built via the System Builder option, where it was embedded in an orthorhombic box of TIP3P waters together with 0.15 M Na^+^ and Cl^−^ ions with a 20 Å solvent buffer. For the permeability study, lipid bilayer systems were built according to the previous 2019 report of Lomize and Pogozheva [27]. Afterwards, the prepared systems were energy-minimized and equilibrated for 10 ns. All compounds’ parameters and topologies were calculated using both the online software Ligand Reader and Modeler (http://www.charmm-gui.org/?doc=input/ligandrm, accessed on 20 January 2021) [28] and the VMD Force Field Toolkit (ffTK) [29]. Binding free energy calculations (Δ*G*) were accomplished with the free energy perturbation (FEP) method and the online software Absolute Ligand Binder [28]. We first prepared the input files and NAMD script with the online-based software CHARMM-GUI Free Energy Calculator (http://www.charmm-gui.org/input/fec, accessed on 20 January 2021) [28]. Afterwards, these inputs were loaded into the NAMD scripts that applied the Charmm-27 force field [30] for simulations, where the equilibration was performed in the NPT ensemble at 300 K and 1 atm (1.01325 bar) with Langevin piston pressure (for ″Complex″ and ″Ligand″) in the presence of a TIP3P water model. Then, 10 ns FEP simulations were performed for each compound, and the last 5 ns of the free energy values were measured for the final free energy values. We used the best binding pose for each compound inside the corresponding protein binding site to investigate their binding stability and mode of interactions. Finally, generated trajectories were visualized and analyzed by the VMD software [29].

### 2.6. Networks Construction

**Figure 3:**
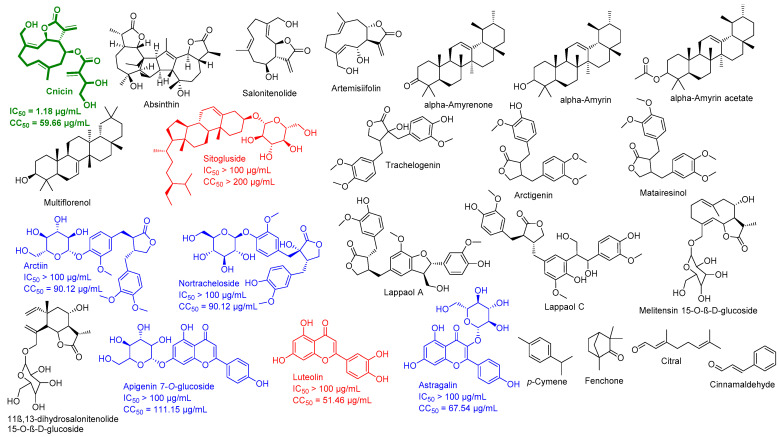
The chemical constituents in the CBE. Blue color indicates the compounds predicted to be active against SARS CoV-2, and green color (i.e., cnicin) indicates the active antiviral hit. Red-colored structures are compounds that did not target any protein in the in silico analysis, and only colored compounds were tested against SARS CoV-2 in vitro based on the results of the primary in silico analysis.

We constructed three networks (Figure 3): (i) a protein–protein interaction (PPI) network that showed the actual binding and interactions between viral–viral and viral–host proteins based on the data obtained from the Swiss-Model repository (https://swissmodel.expasy.org/repository/species/2697049, accessed on 20 January 2021); (ii) a protein-pathway network to indicate the role of each group of proteins in viral pathogenesis (this information was obtained from the KEGG website, the SWISS-Model repository, and a literature survey); and (iii) a compound–protein interaction (CPI) network based on the docking and MDS results. We constructed a connection between *C. benedictus* compounds and both viral and host target proteins if the compound got a docking score of <−7 kcal/mol and remained stable inside the corresponding protein binding site across 150 ns of MDS. All of the above networks were constructed and summarized in a single figure (Figure 3) using the Cytoscape 3.8.2 software (https://www.cytoscape.org/, accessed on 20 January 2021) [31].

### 2.7. In Silico Permeability Studies

Along with the MDS study, we further utilized neural-network-based software study the cellular permeability of the top-hits compounds. The PerMM web server (https://permm.phar.umich.edu/, accessed on 20 January 2021) [32] is a computational tool for assessing the molecules’ passive permeability across lipid bilayers. The applied protocol was dependent on inhomogeneous solubility–diffusion theory using the DOPC bilayer model. PerMM calculates the following parameters: energy profiles along the lipid bilayer, membrane binding affinity, and molecule permeability coefficients. The software’s database currently contains ~500 molecules, (e.g., small synthetic organic compounds and natural products). The ADME properties and their drug-likeness of the top-hits were also calculated using the online website “http://www.swissadme.ch/”( accessed on 20 January 2021) [33].

### 2.8. In Vitro Antiviral Assay

#### 2.8.1. Virus and Cells

DMEM (Dulbecco’s Modified Eagle’s Medium) supplemented with 2% penicillin/streptomycin and 10% FBS was used to maintain Vero-E6 cells at 37 °C and 5% CO_2_. The cells were infected with an hCoV-19/Egypt/NRC-3/2020 isolate at a multiplicity of infection (MOI) of 0.1 in an infection medium (DMEM) supplemented with 1% L-1-tosylamido-2-phenylethyl chloromethyl ketone (TPCK)-treated trypsin, 4% FBS, and 1% penicillin/streptomycin. A fresh infection medium was used after two hours to replace the infection medium containing the virus inoculum and incubated for three days. Cell supernatant was centrifuged at 2500 rpm for 5 min for purification, and the supernatant was then titrated using a plaque assay.

#### 2.8.2. MTT Cytotoxicity Assay

The IC_50_ (half-maximal inhibitory concentration) was determined by preparing stock solutions of the extracts and test compounds in 10% DMSO, which were further diluted with DMEM. Vero-E6 cells with the previously reported MTT method [34] were used to test the cytotoxic activity of the test compounds and extracts. In brief, the cells were plated in 96-well plates and incubated for 24 h at 37 °C in 5% CO_2_ (100 µL/well at a density of 3 × 10^5^ cells/mL). The cells were then treated with different concentrations of extracts/the tested compounds in triplicates. The supernatant was discarded after another 24 h, and cell monolayers were washed three times with sterile 1 × PBS. An MTT solution was added to each well and then incubated at 37 °C for 4 h. The produced formazan crystals were dissolved with 200 µL of acidified isopropanol (0.04 M HCl in absolute isopropanol = 0.073 mL of HCL in 50 mL of isopropanol). Thereafter, the absorbance of formazan solutions was measured at λ_max_ 540 nm using a multi-well plate reader. The following equation was applied to calculate the percentage of cytotoxicity compared to the untreated cells:

$$\%\;\mathrm{cytotoxicity}=\frac{\left(\mathrm{absorbance}\;\mathrm{of}\;\mathrm{cells}\;\mathrm{without}\;\mathrm{treatment}-\mathrm{absorbance}\;\mathrm{of}\;\mathrm{cells}\;\mathrm{with}\;\mathrm{treatment}\right)\times 100}{\mathrm{absorbance}\;\mathrm{of}\;\mathrm{cells}\;\mathrm{without}\;\mathrm{treatment}\;}$$

The produced plot of % cytotoxicity versus sample concentrations was then used to calculate the IC_50_s.

#### 2.8.3. Viral Inhibitory Concentration 50 (IC_50_) Determination

In a humidified 37 °C incubator under a 5% CO_2_ condition, 2.4 × 104 Vero-E6 cells were placed in 96-well plate and then incubated overnight. The cell monolayers were then washed once with PBS and subjected to virus adsorption for 1 h at room temperature. Afterwards, the cells were further overlaid with 50 μL of DMEM mixed with the tested compunds and extracts. The cells were incubated for 72 h, fixed with 4% paraformaldehyde for 20 min, and then stained for 15 min with 0.1% crystal violet. Subsequently, 100 μL of methanol were used to dissolve the crystal violet, and the optical density was measured using Anthos Zenyth 200rt plate reader (Anthos Labtec Instruments, Heerhugowaard, The Netherlands) at 570 nm. The IC_50_ of the compound is the concentration that reduces the virus-induced cytopathic effect (CPE) by 50% relative to a virus control.

#### 2.8.4. Quantitative Real Time Measurement of SARS CoV-2 mRNA Expression

The RNA extraction of the expressed SARS CoV-2 mRNA in infected/treated and untreated cell monolayers was preformed using QIAamp Viral RNA Mini Kit according to manufacturer’s instructions. To assess viral mRNA expression in infected/treated and infected/untreated Vero E6 cells, a TaqMan real-time RT-PCR assay was performed. Briefly, 100 ng of extracted RNA were mixed with TaqMan primers to quantify SARS-CoV-2 mRNA (targeting orf1a: HKU-ORF1b-nsp14F: 5′-TGGGGYTTTACRGGTAACCT-3′, HKU-ORF1b-nsp141P TaqMan Probe: 5′-FAM-TAGTTGTGATGCWATCATGACTAG-TAMRA-3′, HKU-ORF1b-nsp14R: 5′-AACRCGCTTAACAAAGCACTC-3′), together with the other components of Verso 1-step RT-qPCR Kit plus Rox (Invitrogen), in recommended volumes (up to 50 µL per reaction) and thermal protocol conditions.

### 2.9. Statistical Analysis

Data were expressed as mean ± standard error of the mean (SEM) (*n* = 3; three experimental replicates). An ANOVA was applied. Graph Pad Prism was used for statistical calculations (Graph pad Software, San Diego, CA, USA).

## 3. Results

Among the tested plant extracts in our extracts library, the CBE showed the most viral inhibitory activity in vitro (produced inhibition of 73.4% ± 3.2 at a concentration of 10 µg/mL). Consequently, to determine the probable active compounds in the CBE, we subjected all major compounds in this extract to a comprehensive in silico screening. First of all, we reviewed all of the compounds previously reported from *C. benedictus* (Figure 3). We used the Google Scholar, PubMed, and Research Gate search engines using the following keywords “*C. benedictus*, isolated compounds, chemical profiling, and bioactive compounds.”

**Figure 4:**
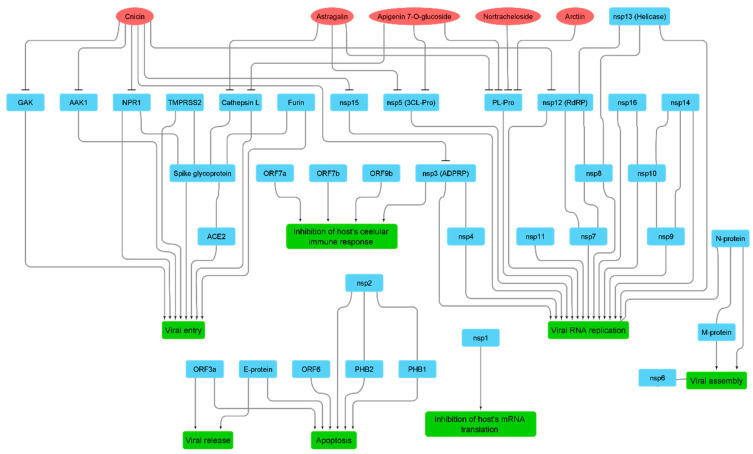
A comprehensive interaction network summarizes our in silico outcomes. Red nodes represent CBE-derived compounds that were predicted to bind with one or more COVID-19-derived proteins. Blue nodes represent all currently available viral and host proteins. Green nodes represent the biological function linked to each group of COVID-19-derived proteins. Black edges represent the interactions between compounds and target proteins, between proteins, and between each group of proteins and their biological function.

Twenty-six compounds belonging to different chemical classes were retrieved in this step. Subsequently, all of these 26 compounds were subjected to an inverse ensemble docking protocol against all the reported SARS CoV-2 proteins (Table S1) to find out the probable molecular targets for each compound. Top hits (a docking score of <−7 kcal/mol) were further subjected to 25 ns of MDS to refine the docking experiments (Table S2). Finally, we only selected compounds that were stable inside the protein binding sites during the simulations (an average RMSD of lower than 5 Å). As a result, we suggested cnicin, apigenin 7-*O*-glucoside, astragalin, arctiin, and nortracheloside to be the main anti-SARS CoV-2 metabolites in the CBE (Figure 3 and Figure 4).

On the other hand, we also reviewed all the viral protein interactions with each other and the human proteins to construct a completed protein–protein interaction (PPI) map for the virus inside the human host cell. For this purpose, we utilized a number of bioinformatics platforms (e.g., SWISS-MODEL; https://swissmodel.expasy.org/repository/species/2697049 (accessed on 28 January 2021) and KEGG; https://www.genome.jp/kegg-bin/show_pathway?map05171+H02398# (accessed on 28 January 2021)) and recent literature to construct this PPI map (Figure 1 and Figure 4). All the represented PPIs in this map are actual reported interactions, and their crystals were deposited in the PDB.

RNA-dependent RNA polymerase (nsp12), nsp10, helicase (nsp13), nsp9, and nsp14 were found to be the most interacting nonstructural proteins (nsps) in the replication complex (1ab) that controls viral replication. However, the spike glycoprotein (S protein) was the most interacting and vital protein involved in viral entry.

As shown in Figure 4, apigenin 7-*O*-glucoside, astragalin, arctiin, and nortracheloside were predicted to interact with and inhibit CL-pro and PL-pro. These two enzymes have an essential role in viral RNA replication because they activate the replication complex (1ab) so that the latter can initiate viral RNA replication [35]. On the other hand, cnicin was found to putatively interact and inhibit six proteins that are involved in viral RNA replication and entry, as well as the inhibition of the host immune response (Figure 1 and Figure 4). The key protein RdRP was among the cnicin targets, thus indicating a direct inhibition of the RNA replication. Cnicin also targeted ADP ribose phosphatase (ADPRP and nsp3), which has dual actions in the viral RNA replication and inhibition of the host innate immunity [36]. Regarding viral entry, cnicin was also found to inhibit neuropilin 1 (NPR-1), which activates the viral S protein to make it ready for subsequent endocytosis [37]. Additionally, it targets both AAK1 and Gak, which have crucial roles in the viral endocytosis process [38].

Our in silico findings highlighted that apigenin 7-*O*-glucoside, astragalin, arctiin, nortracheloside, and (particularly) cnicin were the most probable active components in the CBE that can fight the virus inside the host cell via multiple mechanisms.

### 3.1. Validation of In Silico Analysis

**Figure 5:**
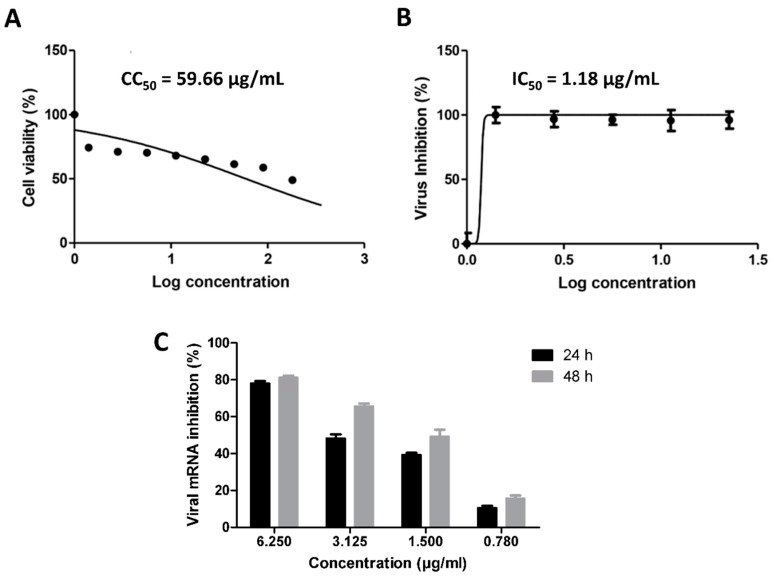
Cytotoxicity and in vitro anti-SARS-CoV-2 activity of cnicin (**A** and **B**, respectively), in addition to viral mRNA% inhibition (**C**). Nonlinear regression analysis of the GraphPad Prism software (version 5.01) was used to calculate the IC_50_ values.

To validate our in silico-derived hits, we evaluated their antiviral activity against the clinical SARS CoV-2 viral isolate in Vero E6 cells. Firstly, the five selected compounds (cnicin, apigenin 7-*O*-glucoside, astragalin, nortracheloside, and arctiin) were evaluated for their cytotoxicity against Vero E6 cells. All of them showed IC_50_ values over 50 µg/mL. Afterwards, the SARS CoV-2-infected Vero E6 cells were incubated with each of the tested compounds at different concentrations. The antiviral activity was then evaluated by determining the viral copy numbers in the cell supernatant via qRT-PCR. Among the predicted compounds, only cnicin exhibited a potential antiviral effect against SARS CoV-2 and showed a dose-dependent inhibition of viral replication, with an IC_50_ of 1.18 µg/mL (Figure 5). Furthermore, the inhibitory effect of cnicin on viral mRNA was measured and normalized to infected and untreated cells. Interestingly, cnicin resulted in significant viral mRNA inhibition at different concentrations at 24 and 48 h post treatment (Figure 5C). The poor antiviral activity of other metabolites (IC_50_ > 100 µg/mL) could be attributed to their high polarity that could hinder permeability across the cell membrane [39]. Accordingly, these findings suggest that that the antiviral activity of the CBE (which produced an inhibition of 73.4% ± 3.2 at a concentration of 10 µg/mL) is likely due to its cnicin content. To further validate our in silico protocol, we tested two of the lowest scoring compounds (luteolin and sitogluside) against SARS CoV-2, and, as predicted, both compounds were inactive (IC_50_ > 100 µg/mL) (Figure 2).

### 3.2. Molecular Interactions Study

Though apigenin 7-*O*-glucoside, astragalin, arctiin, and nortracheloside showed molecular interactions with three targets (Figure 3), they were inactive against in vitro SARS CoV-2 testing. Such outcomes could be attributed to their low permeability across the cell membrane (Figures S1 and S2), which is related to their topological polar surface area (tPSA) and permeability coefficients [27]. Molecules that can cross the cell membrane through passive diffusion usually have tPSA values of less than 140 Å^2^ and permeability coefficients of more than −4 [27,32]. Apigenin 7-*O*-glucoside, astragalin, arctiin, and nortracheloside were found to have high tPSA values ranging from 160.3 to 190.4 Å^2^. Moreover, when subjected to the MDS-based calculation of their permeability coefficients [27,32], all of them showed very low permeability coefficients ranging from −14.17 to −10.8. Such polar glycosylated metabolites are well-known for their poor permeability across cell membrane [39]. Hence, their therapeutic potential is greatly affected by this pharmacokinetic factor.

On the other hand, cnicin, which has a tPSA of 113.3 Å^2^, was able to cross the lipid bilayer (Figures S1 and S2) much more freely, with a permeability coefficient of −3.52. Furthermore, other calculated physicochemical parameters were acceptable according to Lipinski’s and Veber’s rules of drug-likeness [40,41].

**Figure 6:**
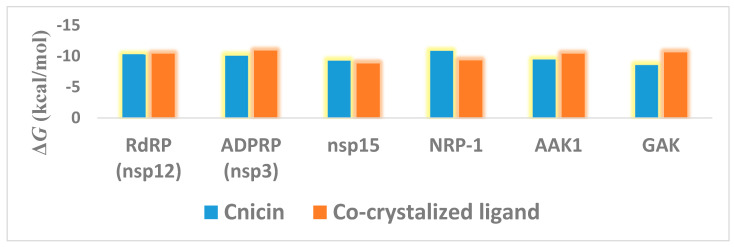
Binding free energies of cnicin inside its target proteins in comparison with the co-crystalized ligand of each protein.

**Figure 7:**
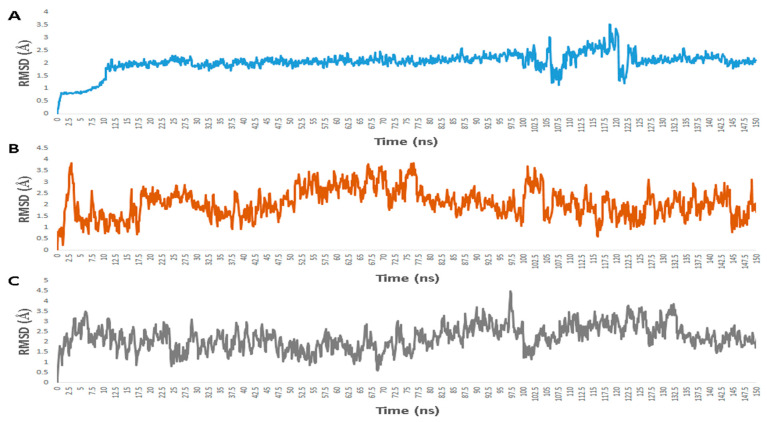
RMSDs of cnicin inside the binding sites of the viral proteins: (**A**) nsp3 (ADPRP), (**B**) nsp12 (RdRP), and (**C**) nsp15.

**Figure 8:**
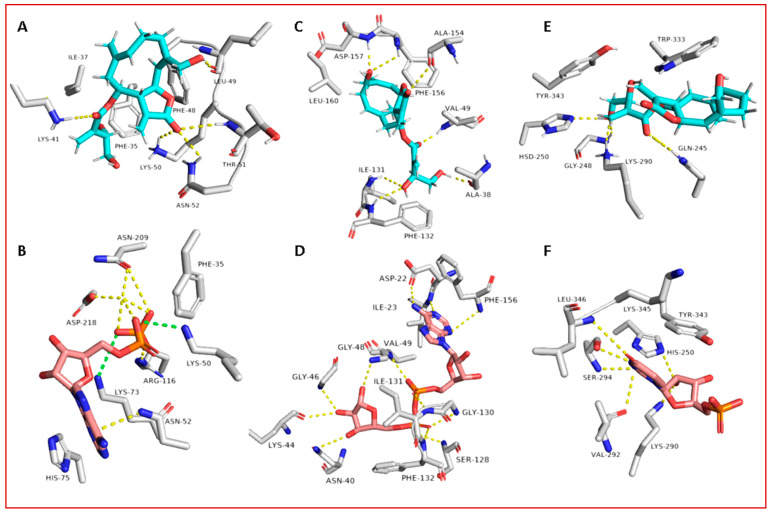
Binding modes of cnicin inside the binding sites of SARS CoV-2 nsp12, nsp3, and nsp15 (**A**, **C**, and **E**, respectively) during 100 ns of MDS (i.e., the last snapshots of the simulations), along with the binding modes of the corresponding co-crystallized ligands (**B**, **D**, and **F**, respectively).

**Figure 9:**
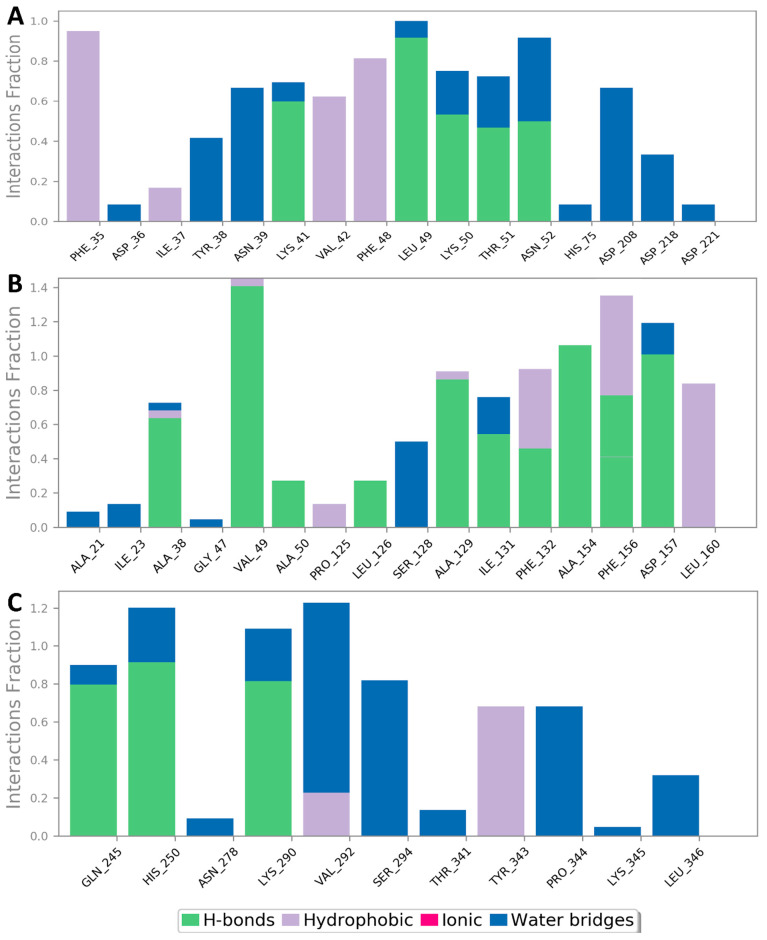
Protein-cnicin contacts inside the viral proteins’ binding sites during 150 ns of MDS: nsp12, nsp3, and nsp15 (**A**, **B**, and **C**, respectively).

Regarding its binding modes inside the active sites of the predicted targets, cnicin got the highest binding free energy (Δ*G* = −10.3 kcal/mol (Figure 6); docking score = −9.7 kcal/mol) against RdRP (i.e., nsp12) at the binding site of the N-terminal nidovirus RdRP-linked nucleotidyltransferase (NiRAN) domain [42]. It achieved a high binding stability during a 150 ns of MDS (RMSD ~ 2.01 Å; see Figure 7) through five strong H-bonds in addition to other three hydrophobic interactions (Figure 8 and Figure 9) with multiple amino acid residues involved in the interaction with the co-crystalized ligand adenine diphosphate (ADP). This binding pocket of the NiRAN domain was recently reported as a promising target for antiviral therapy development [42].

Similarly, cnicin achieved an interesting binding free energy with ADPRP (i.e., nsp3) (Δ*G* = −10.1 kcal/mol (Figure 6); docking score = −9.2 kcal/mol). Additionally, it better-adopted a binding mode (Figure 8) inside the active site than the co-crystalized ligand ADP ribose, where all the molecule’s oxygen atoms were involved in H-bonding and thus had a high stability during the MDS (RMSD ~ 2.08 Å; Figure 7 and Figure 9). Recently, ADPRP was found to have a crucial role in viral replication and interference with the host immune response [36].

Cnicin was also able to achieve a perfect binding affinity toward viral endoribonuclease (nsp15), with a binding free energy lower than that of the co-crystalized ligand (a Δ*G* of −9.3 kcal/mol and a docking score of −9.8 kcal/mol; the co-crystalized ligand got a Δ*G* of −8.8 kcal/mol (Figure 6) and a docking score of −8.6 kcal/mol). Moreover, it was more stable inside the binding site than the co-crystalized ligand during the MDS (RMSD ~ 2.2 Å; Figure 7), and such stability was achieved through three strong H-bonds (<2 Å) and two hydrophobic interactions (Figure 8 and Figure 9). This enzyme proved to have a crucial role in viral replication and, hence, is considered a potential anti-SARS-CoV−2 target [43].

**Figure 10:**
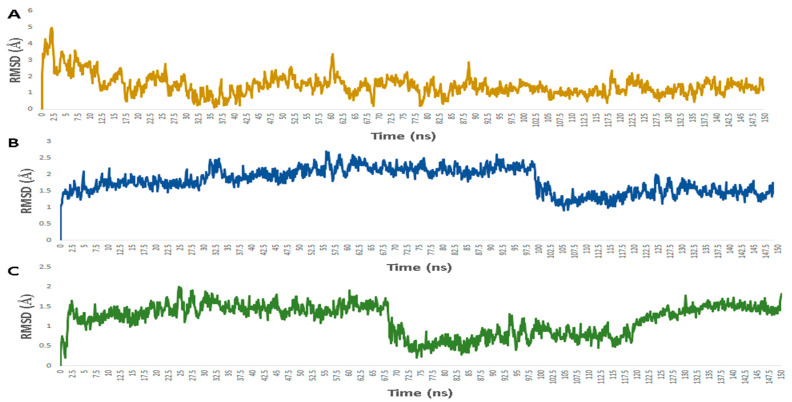
RMSDs of cnicin inside the binding sites of the human proteins: (**A**) NPR, (**B**) GAK, and (**C**) AAK1.

**Figure 11:**
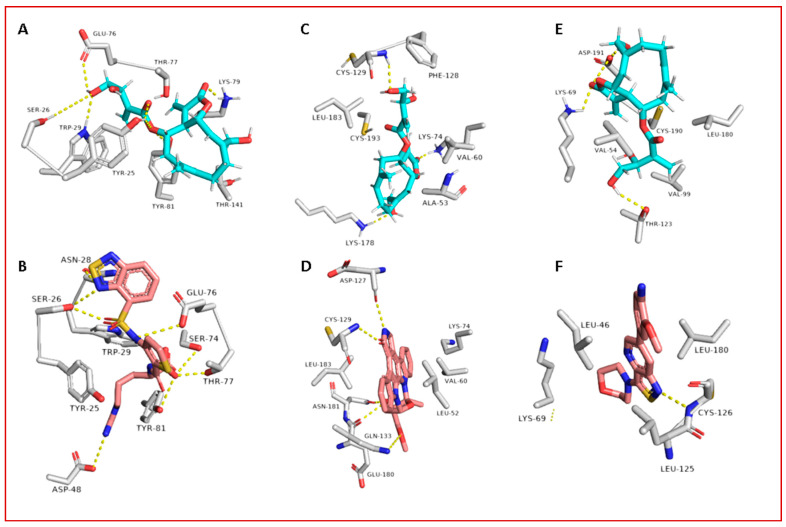
Binding modes of cnicin inside the binding sites of three human targets involved in viral entry—NRP-1, AAK1, and GAK (**A**, **C**, and **E**, respectively)—during 100 ns of MDS (i.e., the last snapshots of the simulations), along with the binding modes of the corresponding co-crystallized ligands (**B**, **D**, and **F**, respectively).

**Figure 12:**
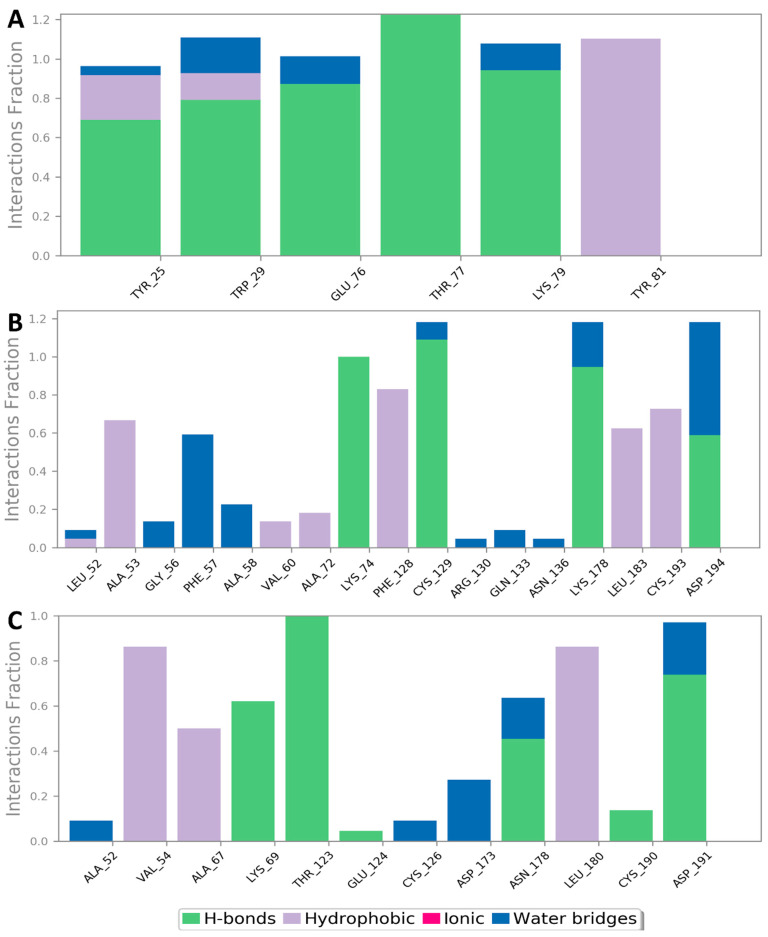
Protein-cnicin contacts inside the human proteins’ binding sites during 150 ns of MDS: NRP-1, AAK1, and GAK (**A**, **B**, and **C**, respectively).

Regarding the human proteins involved in viral entry into the cell, cnicin was able to target three of them. The first one was neuropilin-1 (NRP-1), where it got the highest binding free energy that was even better than the co-crystalized ligand (a Δ*G* of −10.9 (Figure 6) and a docking score of −11.5 kcal/mol; the co-crystalized ligand got a Δ*G* of −9.3 kcal/mol and a docking score of −10.4 kcal/mol). During MDS, cnicin was highly stabilized (RMSD ~ 1.44 Å; Figure 10) inside the binding pocket (B1 domain) through a network of strong H-bonds (<2 Å) in addition to multiple hydrophobic interactions with the molecule’s hydrocarbon body (Figure 11 and Figure 12). NRP-1 is a surface glycoprotein that regulates a number of fundamental processes in carcinogenesis. This protein was recently found to be involved in SARS CoV-2 uptake by epithelial/endothelial cells following S protein cleavage by furin [44,45].

Furthermore, cnicin was able to bind to both adaptor-associated kinase 1 (AAK1) and cyclin G-associated kinase (GAK) (Δ*G* = −9.5 and −8.6 kcal/mol, respectively (Figure 6); docking sores = −9.1 and −8.2 kcal/mol, respectively). These two serine–threonine protein kinases are essential for the SARS CoV-2 endocytosis. Additionally, they regulate intracellular viral operation throughout the assemblage and release of several non-related RNA viruses, e.g., hepatitis C, rabies, and Ebola virus [38]. Cnicin achieved a significant binding stability inside the active sites of both kinases through the course of MDS (RMSD ~ 1.8 and 2.4 Å, respectively; see Figure 11), and binding modes convergent to the co-crystallized inhibitors (Figure 11 and Figure 12).

When taken together with virtual and physics-based modelling experiments, the previous in vitro testing results could suggest that cnicin is a promising SARS CoV-2 multi-target inhibitor with a high potential to be developed into a new antiviral therapeutic agent.

## 4. Discussion

SARS CoV-2 still threatens global health, particularly in developing countries. Moreover, it keeps evolving into new strains with new clinical features [46]. Despite different developed COVID-19 vaccines, the search for new therapeutics is an urgent necessity.

Natural products have been the main pipeline of human medicine throughout history, and they are still able to provide effective therapeutics for our current health crisis. Our continuous screening of small natural products and crude extracts for potential anti-SARS CoV-2 therapeutics led to the discovery of the CBE as a promising candidate.

*C. benedictus* (also known as Blessed thistle) has traditionally been used as a bitter to enhance appetite and digestion. It has also exhibited antimicrobial and anti-inflammatory activities in previous in vitro and in vivo studies [14]. Cnicin is one of the major chemical constituents of Blessed thistle, and it was proven to exhibit a broad antimicrobial activity (e.g., antiparasitic toward *Schistosoma mansoni* and antibacterial) [47,48], but it is not an FDA-approved drug. *C. benedictus* is generally considered safe, and it is used as a component in Benedictine liqueur™ in the USA [14].

With the aid of a number of virtual screening tools (e.g., COVID-19 protein databases, network pharmacology, molecular docking, and molecular dynamic simulations), we constructed a complete interaction network of the viral proteins with each other and human-based proteins involved in cellular entry. Additionally, we suggested a number of *C. benedictus* metabolites to be the active antiviral agents inside its crude extract (CBE) based on our employed in silico protocol: (i) the extensive ensemble docking of all *C. benedictus* previously reported metabolites against all SARS CoV-2 proteins and (ii) the physics-based simulations of all the best hits to further refine the first docking step. Four glycosylated compounds and cnicin were found to interact with nine targets involved in viral replication, entry, and the host cellular immune response. During in vitro testing, cnicin inhibited SARS CoV-2 viral replication in a dose-dependent way, with an IC_50_ of 1.18 µg/mL, but the glycosylated compounds did not. In addition, cnicin showed a considerable safety profile (SI = CC_50_/IC_50_ = 70.3) for further development as an antiviral therapeutic agent.

Further dynamic simulations were conducted to study the molecular interactions of these compounds. The low cellular permeability of the glycosylated constituents appeared to be the main cause of them lacking in vitro activity.

The active compound cnicin was found to possess excellent drug-likeness properties and a high cellular permeability. Additionally, it achieved stable bindings with six protein targets in modes comparable with the previously reported inhibitors, thus suggesting cnicin as an up-and-coming drug candidate for COVID variants.

Ivermectin was the first reported natural product-derived compound that showed promising in vitro inhibitory activity against SARS CoV-2 [49]. Later, it showed promising clinical outcomes [13,50]. Similar to our findings with cnicin in this study, ivermectin exerts its antiviral effect by targeting multiple targets, particularly host-based ones [51]. Currently, we are conducting a comprehensive in vivo study on cnicin as a potential anti-SARS CoV-2 drug, and if we will able to get promising results, we will start a clinical trial on both the plant extract and its major component, cnicin.

## 5. Conclusions

Overall, the present investigation provides another valuable example for exploring bioactive plant-derived natural products. Moreover, it highlights the power of integrating in silico tools with in vitro testing in accelerating the rate of discovery of novel therapeutics, particularly during crises like our current viral pandemic. Our virtual and physics-based modelling experiments, together with the promising in vitro testing results, shed light on cnicin as a promising SARS CoV-2 multi-target inhibitor with a high potential to be developed into a new antiviral therapeutic agent. Further in vivo and subsequent clinical trials are underway to comprehensively evaluate cnicin and the *C. benedictus* extract as promising therapeutic agents against COVID-19.

## Supplementary Materials

The following are available online at https://www.mdpi.com/article/10.3390/antibiotics10050542/s1: Figure S1: Transfer energies of CBE’s top-scoring compound across the membrane bilayer; Figure S2: Cnicin positions during its transfer across the lipid bilayer; Table S1: All SARS CoV-2-related proteins used in this study; Table S2: Compounds that got docking scores <−7 kcal/mol and were stable during the course of 25 ns molecular dynamic simulations.
