# Supplementary material for: Cnicin as an Anti-SARS-CoV-2: An Integrated In Silico and In Vitro Approach for the Rapid Identification of Potential COVID-19 Therapeutics
Source: Antibiotics (Basel). 2021 May 7;10(5):542. doi: 10.3390/antibiotics10050542 (PMC8150330; doi:10.3390/antibiotics10050542)
Supplement: Supplementary file 1 [file antibiotics-10-00542-s001.zip › antibiotics-1213153-supplementary.pdf]

Article

Cnicin as an Anti-SARS-CoV-2: An Integrated in Silico and in Vitro Approach for the Rapid Identification of Potential COVID-19 Therapeutics

Hani A. Alhadrami [1,2,†], Ahmed M. Sayed [3,†], Hossam M. Hassan [3,4], Khayrya A. Youssif [5], Yasser Gaber [6,7], Yassmin Moatasim [8], Omnia Kutkat [8], Ahmed Mostafa [8], Mohamed Ahmed Ali [8], Mostafa E. Rateb [9,†], Usama Ramadan Abdelmohsen [10,11,*] and Noha M. Gamaleldin [12,*]

[1] Department of Medical Laboratory Technology, Faculty of Applied Medical Sciences, King Abdulaziz University, P.O. BOX 80402, Jeddah 21589, Saudi Arabia; hanialhadrami@kau.edu.sa
[2] Molecular Diagnostic Lab, King Abdulaziz University Hospital, King Abdulaziz University, P.O. BOX 80402, Jeddah 21589, Saudi Arabia
[3] Department of Pharmacognosy, Faculty of Pharmacy, Nahda University, Beni-Suef 62513, Egypt; Ahmed.mohamed.sayed@nub.edu.eg (A.M.S.); hossam.mokhtar@nub.edu.eg (H.M.H.)
[4] Department of Pharmacognosy, Faculty of Pharmacy, Beni-Suef University, Beni-Suef 62513, Egypt; hossam.mokhtar@nub.edu.eg
[5] Department of Pharmacognosy, Faculty of Pharmacy, Modern University for Technology and Information, Cairo 11865, Egypt; khayrya.youssif@gmail.com
[6] Department of Microbiology and Immunology, Faculty of Pharmacy, Beni-Suef University, Beni-Suef 62511, Egypt; yasser.gaber@pharm.bsu.edu.eg
[7] Department of Pharmaceutics and Pharmaceutical Technology, Faculty of Pharmacy, Mutah University, Karak 61710, Jordan
[8] Center of Scientific Excellence for Influenza Virus, Environmental Research Division, National Research Centre, Giza 12622, Egypt; Yasmin.Moatasim@human-link.org (Y.M.); Omnia.Abdelaziz@human-link.org (O.K.); ahmed_elsayed@daad-alumni.de (A.M.); mohamedahmedali2004@yahoo.com (M.A.A.)
[9] School of Computing, Engineering & Physical Sciences, University of the West of Scotland, Paisley PA1 2BE, UK; Mostafa.Rateb@uws.ac.uk
[10] Department of Pharmacognosy, Faculty of Pharmacy, Deraya University, New Minia 61111, Egypt
[11] Department of Pharmacognosy, Faculty of Pharmacy, Minia University, Minia 61519, Egypt,
[12] Department of Microbiology, Faculty of Pharmacy, The British University in Egypt (BUE), Cairo 11837, Egypt
† These authors contributed equally to this work.
* Correspondence: usama.ramadan@mu.edu.eg (U.R.A.); noha.gamaleldin@bue.edu.eg (N.M.G.)

Table S1. All SARS CoV-2-related proteins used in this study.

Protein	Name	Function	PDB Code	Docking box co-ordinates
		**Viral Proteins (Nonstructural proteins)**		
nsp1*	-	Host translation inhibitor	7K3N	-
nsp2*	-	Play a role in the modulation of host cell survival signaling pathway by interacting with host PHB and PHB2	Deposited model in Swiss-Model	-
nsp3	ADP ribose phosphatase	Activate replicase polyprotein	6W02	X= -0.66<br>Y= -8.97<br>Z= -22.42
nsp4*	-	Participates in the assembly of virally-induced cytoplasmic double-membrane vesicles necessary for viral replication	Deposited model in Swiss-Model	-
nsp5 (3CL-Pro)	Main protease	Cleaves the C-terminus of replicase polyprotein at 11 sites	6lu7	X= 12.87<br>Y= 16.13<br>Z= 68.64
nsp6*	-	Plays a role in the initial induction of autophagosomes	Deposited model in Swiss-Model	-
nsp7*	-	Plays a role in viral RNA synthesis	6M5I	-
nsp8*	-	Plays a role in viral RNA synthesis	6M5I	-
nsp9*	-	May participate in viral replication by acting as a ssRNA-binding protein	6WC1	-

nsp10*	-	plays an essential role in viral mRNAs cap methylation	6ZPE	-
nsp11 (PL-Pro)	Papain-like protease	Participate in the activation of the replicase polyprotein	6WX4	X= 10.81<br>Y= -28.52<br>Z= -41.4
nsp12 (Pol; RdRP)	RNA-directed RNA polymerase	Responsible for replication and transcription of the viral RNA genome	6XEZ	X= 139.42<br>Y= 201.96<br>Z= 135.29
nsp13 (Hel)	Helicase	Displaying RNA and DNA duplex-unwinding activities with 5' to 3' polarity. Activity of helicase is dependent on magnesium	6XEZ	X= 139.42<br>Y= 201.96<br>Z= 135.29
nsp14 (ExoN)*	Proofreading exoribonuclease	An exoribonuclease activity acting on both ssRNA and dsRNA in a 3' to 5' direction	Deposited model in Swiss-Model	-
Nsp15 (NendoU)	Uridylate-specific endoribonuclease	Mn(2+)-dependent, uridylate-specific enzyme, which leaves 2'-3'-cyclic phosphates 5' to the cleaved bond.	5S6X	X= 64.14<br>Y= -71.5<br>Z= 25.29
nsp16	2'-*O*-methyltransferase	Methyltransferase that mediates mRNA cap 2'-O-ribose methylation to the 5'-cap structure of viral mRNAs	6W4H	X= 84.62<br>Y= 15.56<br>Z= 26.39
**Viral Proteins (Structural Proteins)**				
S glycoprotein	Spike glycoprotein	Binding to human ACE2 receptor and internalization of the virus into the endosomes of the host cell induces conformational changes in the Spike glycoprotein	6lzg	X= -36.69<br>Y= 39.28<br>Z= 14.30
E-protein	Envelope small membrane protein	Plays a central role in virus morphogenesis and assembly	7k3g	X= 10.13<br>Y=0.12<br>Z= 0.71
M-protein*	Membrane protein	Component of the viral envelope that plays a central role in virus morphogenesis and assembly via its interactions with other viral proteins	Deposited model in Swiss-Model	-
N-protein*	Nucleoprotein	Packages the positive strand viral genome RNA into a helical ribonucleocapsid (RNP) and plays a fundamental role during virion assembly through its interactions with the viral genome and membrane protein M	6M3M	-
**Viral proteins (Accessory Proteins)**				
ORF3a*	ORF3a protein	Forms homotetrameric potassium sensitive ion channels (viroporin) and may modulate virus release	6XDC	-
ORF6*	ORF6 protein	Disrupts cell nuclear import complex formation by tethering karyopherin alpha 2 and karyopherin beta 1 to the membrane	Deposited model in Swiss-Model	-
ORF7a*	ORF7a protein	Plays a role as antagonist of host tetherin (BST2), disrupting its antiviral effect	6W37	-
ORF7b	ORF7b protein	-	-	-
ORF8*	ORF8 protein	May play a role in host-virus interaction	7JTL	-
ORF9b	ORF9b protein	Plays a role in the inhibition of host innate immune response by targeting the mitochondrial-associated adapter MAVS	6Z4U	X= 10.24<br>Y= -2.84<br>Z= -1.89
**Human-derived Proteins**				
ACE2	Angitensin converting enzyme 2	Responsible for attachment of SARS CoV	6LZG	X= -30.8<br>Y= 12.73<br>Z= -3.63
Furin	Furin	Activation of S-protein	6EQX	X= 43.99<br>Y= -39.11<br>Z= -5.55
TMPRSS2*	Transmembrane protease, serine 2	Activation of S-protein	Deposited model in Swiss-Model	-
CatL	Cathepsin L	Activation of S-protein	2YJC	X= 8.92<br>Y= 36.19<br>Z= 19.45
NPR1	Neuropilin 1	Facilitates viral entry	3I97	X= 0.21

				Y= -3.08<br>Z= -9.95
PHB1*	Prohibitin subunit1	In the mitochondria, together with PHB2, forms large ring complexes (prohibitin complexes) in the inner mitochondrial membrane (IMM) and functions as chaperone protein that stabilizes mitochondrial respiratory enzymes and maintains mitochondrial integrity	Deposited model in Swiss-Model	-
PHB2*	Prohibitin subunit2	In the mitochondria, together with PHB2, forms large ring complexes (prohibitin complexes) in the inner mitochondrial membrane (IMM) and functions as chaperone protein that stabilizes mitochondrial respiratory enzymes and maintains mitochondrial integrity	Deposited model in Swiss-Model	-
AAK1	Adaptor Protein 2 Associated Kinase	Participate in viral endocytosis	4WSQ	X= 8.44<br>Y= -13.38<br>Z= -52.35
GAK	Cyclin-G associated kinase	Participate in viral endocytosis	4y8d	X= 30.39<br>Y= 47.92<br>Z= -59.73

*These protein structures are either calculated models (deposited in SWISS-model website) or have not co-crystalized ligand. In this case docking experiments were performed on all possible binding cavities on the protein. This was achieved by enclosing the whole protein inside the docking box during the preparation of docking experiments. .

Table S2. Compounds that got docking scores< -7 kcal/mol and were stable during the course of 25 ns molecular dynamic simulations (got an average RMSD < 5 Å).

	Viral proteins									
**Compound**	**3CL-Pro**		**PL-Pro**		**nsp3**		**nsp12**		**nsp15**	
	**Docking score**	**Average RMSD**	**Docking score**	Average RMSD	Docking score	Average RMSD	Docking score	Average RMSD	Docking score	Average RMSD
**Cnicin**	> -7	> 5	> -7	> 5	**-9.2**	**2.08**	**-9.7**	**2.01**	**-9.8**	**2.2**
**Apigenin 7-*O*-glucoside**	**-9.2**	**1.9**	**-8.6**	**2.6**	> -7	> 5	> -7	> 5	> -7	> 5
**Astragalin**	**-9.5**	**2.1**	**-8.3**	**2.9**	> -7	> 5	> -7	> 5	> -7	> 5
**Arctiin**	> -7	> 5	**-7.3**	**3.9**	> -7	> 5	> -7	> 5	> -7	> 5
**Nortracheloside**	> -7	> 5	**-7.1**	**-3.5**	> -7	> 5	> -7	> 5	> -7	> 5
**Sitogluside***	> -7	> 5	> -7	> 5	> -7	> 5	> -7	> 5	> -7	> 5
**Luteolin****	**-7.5**	> 5	**-7.1**	> 5	> -7	> 5	> -7	> 5	> -7	> 5

	Non-viral proteins							
	**Cathepsin L**		**NPR1**		**AAK1**		**GAK**	
	Docking score	Average RMSD	Docking score	Average RMSD	Docking score	Average RMSD	Docking score	Average RMSD
**Cnicin**	> -7	> 5	**-11.5**	**1.44**	**-9.1**	**1.8**	**-8.2**	**2.4**
**Apigenin 7-*O*-glucoside**	**-7.9**	**3.4**	> -7	> 5	> -7	> 5	> -7	> 5
**Astragalin**	**-8.1**	**2.2**	> -7	> 5	> -7	> 5	> -7	> 5
**Arctiin**	> -7	> 5	> -7	> 5	> -7	> 5	> -7	> 5
**Nortracheloside**	> -7	> 5	> -7	> 5	> -7	> 5	> -7	> 5
**Sitogluside***	> -7	> 5	> -7	> 5	> -7	> 5	> -7	> 5
**Luteolin****	**-7.0**	> 5	> -7	> 5	> -7	> 5	> -7	> 5

*This compounds achieved docking scores >-7 and unstable bindings (RMSD > 5Å) with all proteins.**This compound got docking score <-7 kcal/mol with some proteins, but was unstable upon molecular dynamic simulation (got RMSD > 5Å).These two compounds (i.e. sitogluside and luteolin) were selected to validate our in-silico approach. They were inactive upon in-vitro viral inhibitory testing (got IC_{50} > 100 μg/mL).

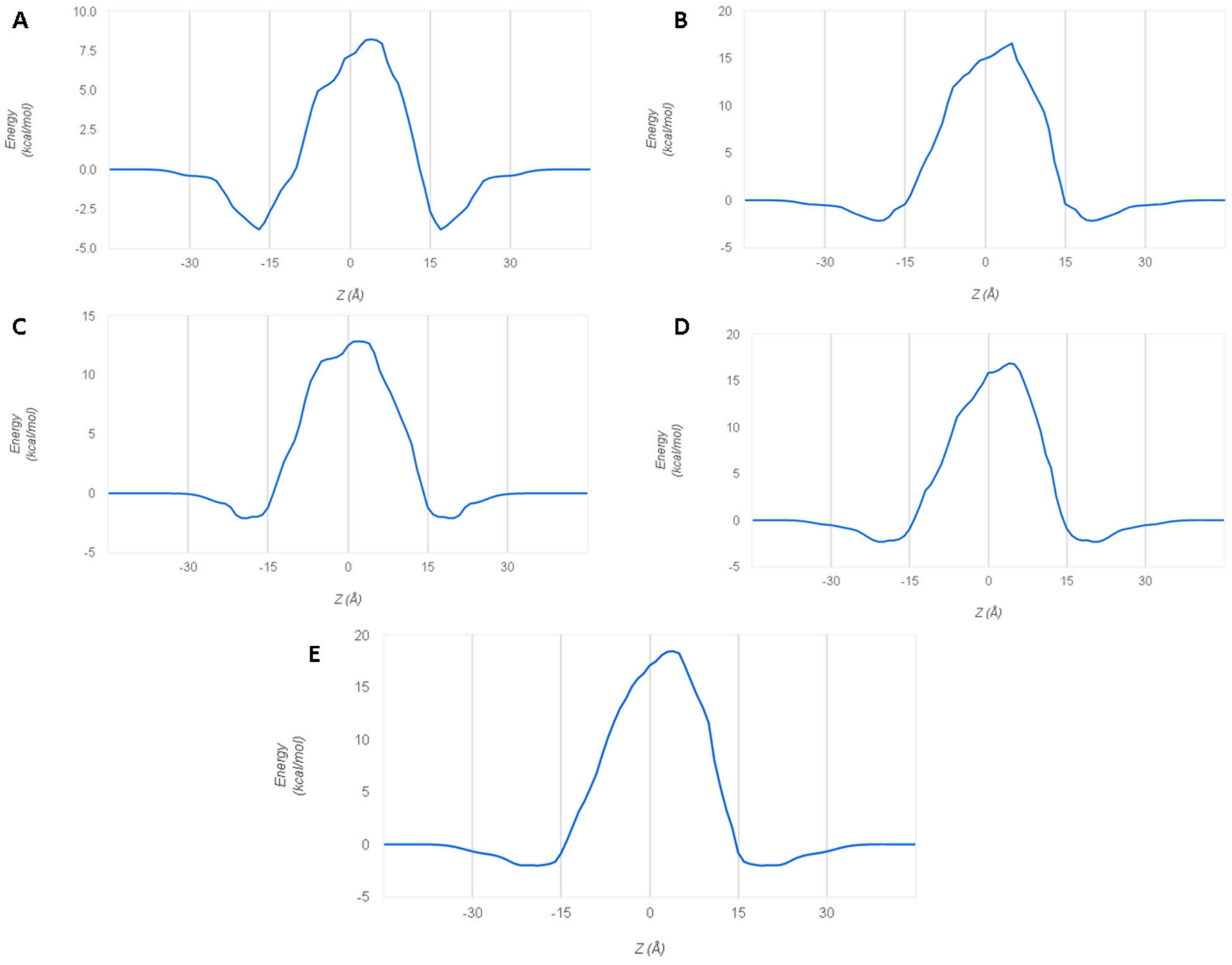

Figure S1. Transfer energies of CBE's top-scoring compound across the membrane bilayer (DOPC bilayer): (A) cnicin, (B) nortracheloside, (C) arctiin, (D) apigenin 7-O-glucoside, (E) astragalin.

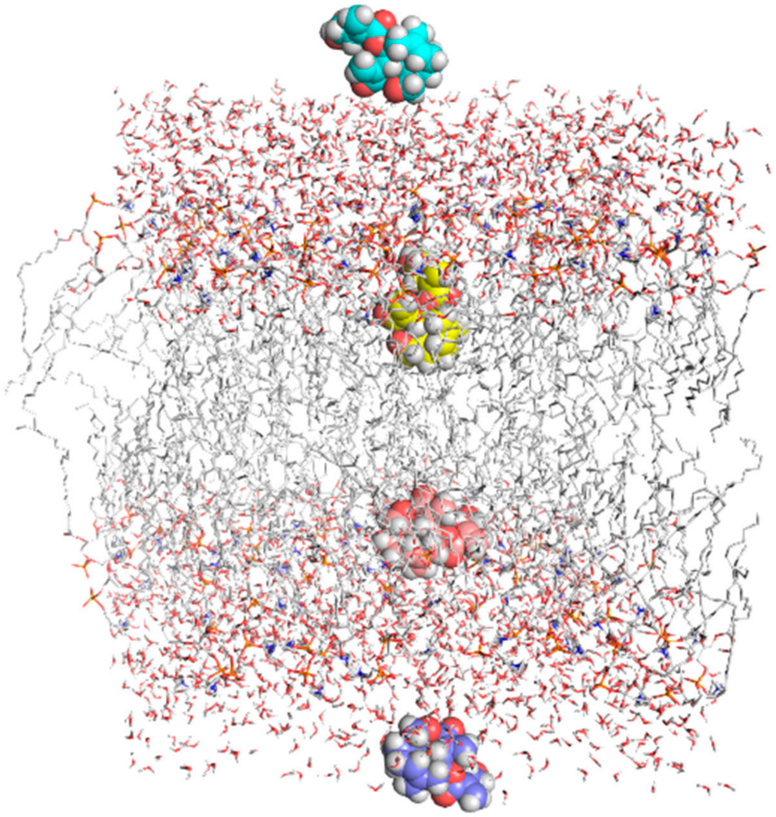

Figure S2. Cnicin positions during its transfer across the lipid bilayer.
